# Increased reactive oxygen species and exhaustion of quiescent CD34-positive bone marrow cells may contribute to poor graft function after allotransplants

**DOI:** 10.18632/oncotarget.8810

**Published:** 2016-04-18

**Authors:** Yuan Kong, Yang Song, Yue Hu, Min-Min Shi, Yu-Tong Wang, Yu Wang, Xiao-Hui Zhang, Lan-Ping Xu, Kai-Yan Liu, Hong-Kui Deng, Xiao-Jun Huang

**Affiliations:** ^1^ Peking University People's Hospital, Peking University Institute of Hematology, Beijing Key Laboratory of Hematopoietic Stem Cell Transplantation, Collaborative Innovation Center of Hematology, Peking University, Beijing, China; ^2^ Peking-Tsinghua Center for Life Sciences, Academy for Advanced Interdisciplinary Studies, Peking University, Beijing, China; ^3^ Key Laboratory of Cell Proliferation and Differentiation of the Ministry of Education, School of Life Sciences, Peking University, Beijing, China

**Keywords:** poor graft function, allotransplant, haematopoietic stem cells, reactive oxygen species

## Abstract

Poor graft function (PGF) is a fatal complication following allogeneic haematopoietic stem cell transplantation. However, the underlying mechanism is unclear. Effective cross-talk between haematopoietic stem cells (HSCs) and bone marrow microenvironment is important for normal haematopoiesis. Normal HSCs reside in a hypoxic bone marrow microenvironment that protects them from oxidative stress that would otherwise inhibit their self-renewal and results in bone marrow failure. Whether an increased level of reactive oxygen species (ROS) causes PGF following allotransplant is unclear. Using a prospective case-pair study, we identified increased levels of ROS in CD34^+^ bone marrow cells in subjects with PGF. Elevated ROS levels was associated with an increased frequency of DNA strand breaks, apoptosis, exhaustion of quiescent CD34^+^ cells and defective colony-forming unit plating efficiency, particularly in the CD34^+^CD38^−^ fraction. Up-regulated intracellular p53, p21, caspase-3 and caspase-9 levels (but not p38) were detected in CD34^+^ cells, particularly in the CD34^+^CD38^−^ fraction. To further study the potential role of ROS levels in post-transplant haematopoiesis, CD34^+^ bone marrow cells from subjects with good graft function were treated with H_2_O_2_. This increased ROS levels resulting in defective CD34^+^ cells, an effect partially reversed by N-acetyl-L-cysteine. Moreover, CD34^+^ bone marrow cells from the donors to subjects with poor or good graft function exhibited comparable haematopoietic reconstitution capacities in the xeno-transplanted NOD-Prkdc^scid^IL2rg^null^ mice. Thus, even if the transplanted donors' bone marrow CD34^+^ cells are functionally normal pre-transplant, ROS-induced apoptosis may contribute to the exhaustion of CD34^+^ bone marrow cells in subjects with PGF following allotransplant.

## INTRODUCTION

Poor graft function is an important, often fatal complication following allogeneic haematopoietic stem cell transplant [1–4]. The etiology of poor graft function is complex and includes many factors such as bone marrow toxic drugs, infections, graft-*versus*-host disease (G*v*HD) and an impaired bone marrow microenvironment [2, 3, 5–9]. One or more of these factors may operate in different persons or the same person with poor graft function post-allotransplant.

Interactions between haematopoietic stem and progenitor cells and the bone marrow microenvironment are important in maintaining normal haematopoiesis [10–13]. We recently reported that the transplanted donor CD34-positive cells were quantitatively normal in subjects with poor graft function, but the frequency of bone marrow CD34-positive cells were dramatically reduced and bone marrow endosteal, vascular microenvironment were impaired in subjects with poor graft function compared with those with good graft function posttransplant [2, 3], raising the question whether bone marrow CD34-positive cells in subjects with poor graft function are functionally impaired posttransplant, or the transplanted donors' CD34-positive cells are already defective pretransplant. Moreover, what drives the bone marrow CD34-positive cells impairing functionally posttransplant and its molecular mechanisms remain to be elucidated in poor graft function.

Reactive oxygen species (ROS) are free radicals derived from diatomic oxygen and exhibit diverse reactivities. ROS affect cell cycle progression, cell motility and growth factor signaling in many cell types, including haematopoietic stem and progenitor cells [14, 15]. Haematopoietic stem and progenitor cells may occupy a hypoxic niche in the bone marrow microenvironment that protects them from oxidative stress [16, 17]. In Atm^−^ or FoxO^−^ deficient mice, haematopoietic stem cells are depleted due to increased ROS levels. This effect is reversible by treatment with the anti-oxidative drug N-acetyl-L-cysteine [18]. We hypothesized that increased levels of ROS in the bone marrow microenvironment post-allotransplant result in the depletion of donor haematopoietic stem and progenitor cells, leading to poor graft function.

## RESULTS

### Reduced quiescent cells and increased levels of DNA double-strand breaks and apoptosis in CD34-positive bone marrow cells from subjects with poor graft function

**Figure 1:**
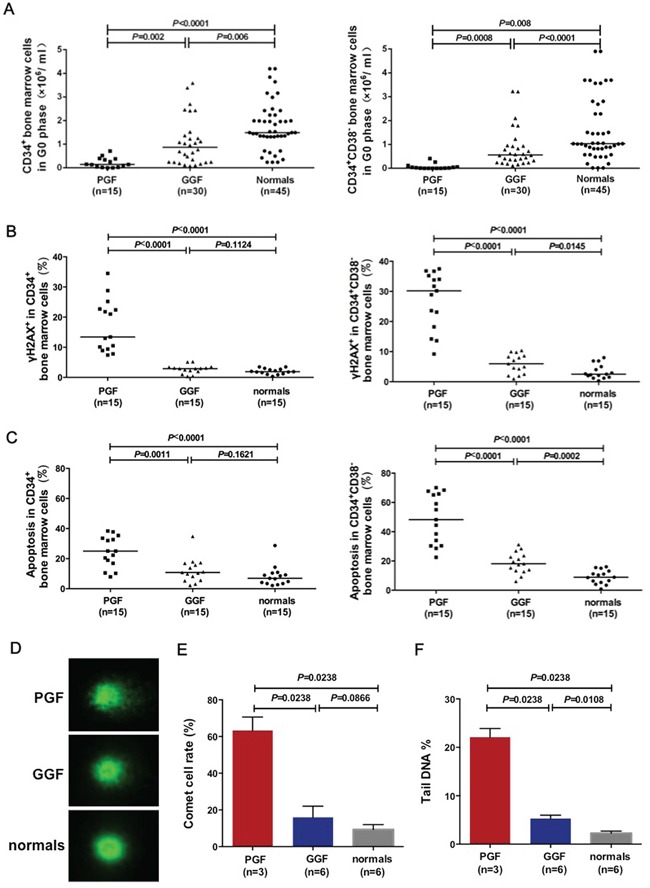
Fraction of CD34-positive quiescent cells, DNA double-strand breaks and apoptosis Reduced quiescent cells **A.** but increased levels of DNA double-strand breaks **B.** and apoptosis **C.** were observed in bone marrow CD34-positive cells and CD34-positive, CD38-negative cells from subjects with poor graft function. Representative images of comet assay were shown in CD34-positive cells from normals and subjects with poor or good graft function **D.** Increased percentages of the comet cells **E.** and the tail DNA **F.** were observed in bone marrow CD34-positive cells from subjects with poor graft function.

Subjects with poor or good graft function were evaluated at comparable intervals posttransplant to minimize bias. Subjects with poor graft function had significantly lower numbers of CD34-positive cells (Figure 1A; 0.21 ± 0.06×10E+6 *vs.* 1.09±0.18×10E+6; *P*=0.002) and CD34-positive, CD38-negative cells (Figure 1A; 0.07±0.03×10E+6 *vs*. 0.84±0.15×10E+6; *P*=0.0008) in the G_0_ phase compared with subjects with good graft function. Subjects with good graft function had significantly lower numbers of CD34-positive cells (Figure 1A; 1.09±0.18×10E+6 *vs.* 1.76±0.15×10E+6; *P*=0.006) and CD34-positive, CD38-negative cells (Figure 1A; 0.84±0.15×10E+6 *vs*. 1.57±0.20×10E+6; *P*<0.0001) in G_0_ phase compared with normals.

Subjects with poor graft function had a higher frequency of γ-H2AX-positive DNA double-strand breaks in CD34-positive bone marrow cells than subjects with good graft function (Figure 1B, 17.13%±2.22% *vs.* 2.71%±0.37%; *P*<0.0001). The frequency of γ-H2AX-positive DNA double-strand breaks was even higher in the quiescent CD34-positive, CD38-negative fraction (Figure 1B, 27.11%±2.45% *vs.* 5.97%±0.83%; *P*<0.0001).

Apoptosis was also markedly increased in CD34-positive bone marrow cells from subjects with poor graft function (Figure 1C, 24.48%±2.66% *vs.* 12.19%±2.08%; *P*=0.001), particularly in the CD34-positive, CD38-negative fraction (Figure 1C, 48.55%±4.38% *vs.* 18.65%±1.85%; *P*<0.0001).

Comet assay was performed to confirm the DNA damage of bone marrow CD34-positive cells. As shown in Figure 1D, the bone marrow CD34-positive cells from normals are round-shaped without tails. By contrast, the CD34-positive cells from subjects with poor graft function showed longer tails than those with good graft function. Moreover, both the frequencies of comet cells and tail DNA in bone marrow CD34-positive cells of subjects with poor graft function were significantly higher than those with good graft function and normals (Figure 1E, 1F).

### Defective CFU plating-efficiency of CD34-positive bone marrow cells from subjects with poor graft function

**Figure 2:**
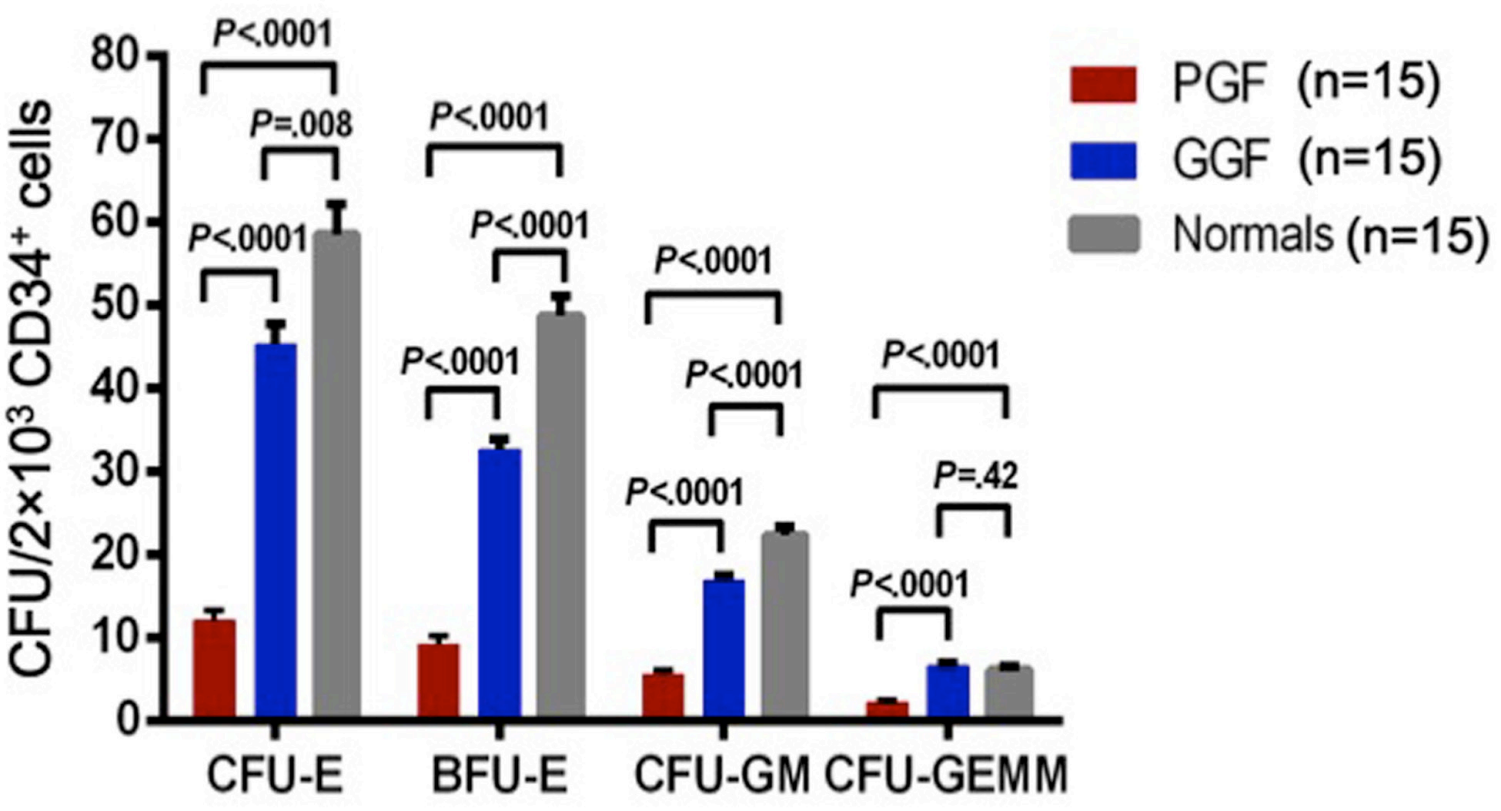
Defective colony-forming unit plating efficiency of CD34-positive bone marrow cells from subjects with poor graft function compared to those with good graft function Colony-forming unit erythroid (CFU-E), burst-forming unit erythroid (BFU-E), colony-forming unit granulocyte–macrophages (CFU-GM), and colony-forming unit-granulocyte, -erythroid, -macrophage and -megakaryocyte (CFU-GEMM) were scored using an inverted light microscope.

CD34-positive bone marrow cells from subjects with poor graft function had significantly lower plating efficiencies (CFU counts/2×10E+3 CD34-positive cells; Figure 2) for all type of haematopoietic progenitors, including CFU-E (12±1 *vs*. 45±3; *P*<0.0001), BFU-E (9±1 *vs.* 33±1; *P*<0.0001), CFU-GM (6±1 *vs*. 17±1; *P*<0.0001) and CFU-GEMM (2±0.3 *vs*. 7±0.4; *P*<0.0001), compared to subjects with good graft function. The CFU plating efficiencies of the subjects with good graft function were lower than those of normals (CFU-E 45±3 *vs*. 59±4; *P*=0.008; BFU-E 33±1 *vs.* 49±2; *P*<0.0001; CFU-GM 17±1 *vs*. 22±1; *P*<0.0001; and CFU-GEMM 7±0.4 *vs*. 6±0.4; *P*=0.42).

### Increased levels of ROS and p53 are associated with exhaustion of CD34-positive bone marrow cells

**Figure 3:**
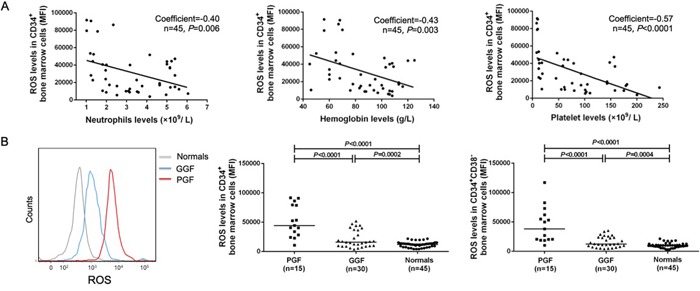
Elevated levels of ROS in subjects with poor graft function **A.** Inverse correlations were observed between hematological parameters (including neutrophil, hemoglobin and platelet) and ROS levels in CD34-positive bone marrow cells of subjects post-transplant. **B.** Increased levels of ROS in bone marrow CD34-positive cells, particularly in the CD38-negative fraction, in transplant recipients with poor graft function post-transplant.

Intracellular levels of ROS, p53, phospho-p53, p21, phospho-p38, caspase-3 and caspase-9 were analyzed in subjects with poor or good graft function and normals. Inverse correlations between subject neutrophil levels posttransplant and ROS levels in CD34-positive bone marrow cells (Pearson r=−0.40 [95% CI −0.62, −0.12]; *P*=0.006; n=45), between hemoglobin concentrations and ROS levels in CD34-positive bone marrow cells (Pearson r=−0.43 [95% CI −0.64, −0.15]; *P*=0.003; n=45) and between platelet levels and ROS levels in CD34-positive bone marrow cells (Pearson r=−0.57 [95% CI] −0.73, −0.31); *P*<0.0001; n=45) were observed (Figure 3A).

Subjects with poor graft function had significantly higher intracellular ROS levels (Figure 3B) in CD34-positive bone marrow cells (51820±7001 *vs.* 21018±2655; *P*<0.0001), particularly in CD34-positive, CD38-negative cells (44475±7752 *vs.* 15756±1686; *P*<0.0001), than subjects with good graft function. Subjects with good graft function had significantly higher ROS levels in CD34-positive bone marrow cells (21018±2655 *vs.* 11911±699; *P*=0.0002) and CD34-positive, CD38-negative cells (15756±1686 *vs.* 9896±619, *P*=0.0004) compared with normals.

**Figure 4:**
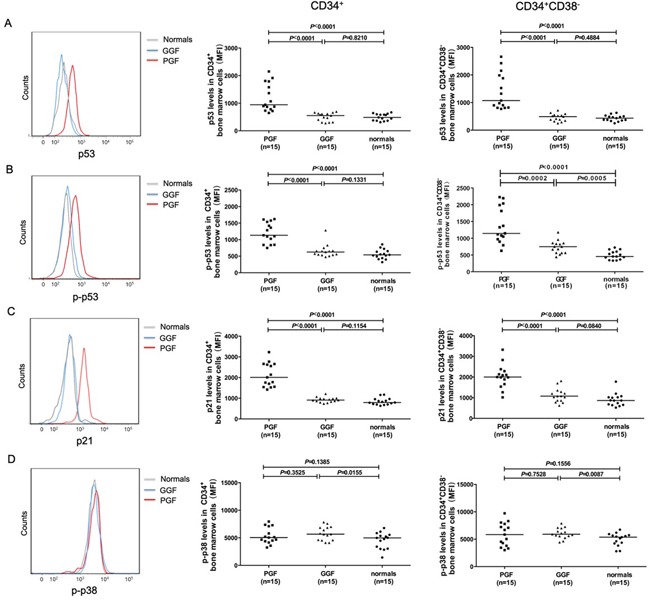
Elevated levels of intracellular p53, phospho-p53, and p21 (but not phospho-p38) in subjects with poor graft function Up-regulated intracellular p53 **A.** phospho-p53 **B.** and p21 **C.** were detected in CD34-positive cells, particularly in the CD38-negative fraction in transplant recipients with poor graft function post-transplant. **D.** Similar intracellular levels of phospho-p38 in bone marrow CD34-positive cells, particularly in the CD38-negative fraction, in transplant recipients with good or poor graft function.

**Figure 5:**
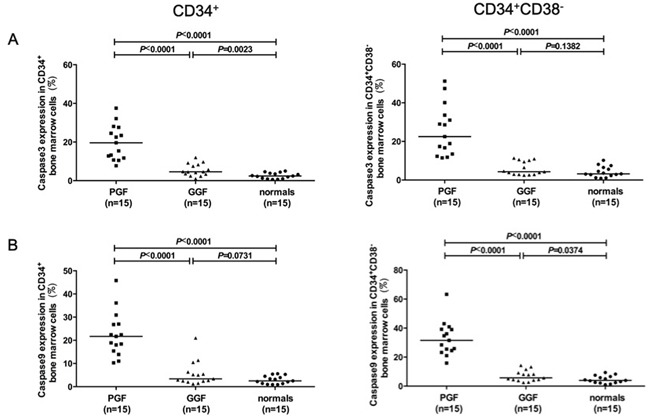
Elevated levels of intracellular caspase-3, and caspase-9 in subjects with poor graft function Up-regulated intracellular caspase-3 **A.** and caspase-9 **B.** were detected in bone marrow CD34-positive cells, particularly in the CD38-negative fraction in subjects with poor graft function post-transplant.

Subjects with poor graft function had markedly increased levels of p53 (Figure 4A; 1209±132 *vs*. 504±39; *P*<0.0001), phospho-p53 (Figure 4B; 1202±79.63 *vs.* 657.0±50.88; *P*<0.0001), p21 (Figure 4C; 2128±148 *vs.* 925±32; *P*<0.0001), caspase-3 (Figure 5A; 19.82%±2.32% *vs.* 5.54%±0.81%; *P*<0.0001) and caspase-9 (Figure 5B; 22.60%±2.48% *vs.* 5.56%±1.36%; *P*<0.0001) compared to subjects with good graft function. By contrast, both cohorts had similar intracellular levels of phospho-p38 (Figure 4D; 5337±376 *vs.* 5805±322; *P*=0.35).

### H_2_O_2_-induced increases in ROS that result in defective CD34-positive bone marrow cells from subjects with good graft function are partially reversed by N-acetyl-L-cysteine

**Figure 6:**
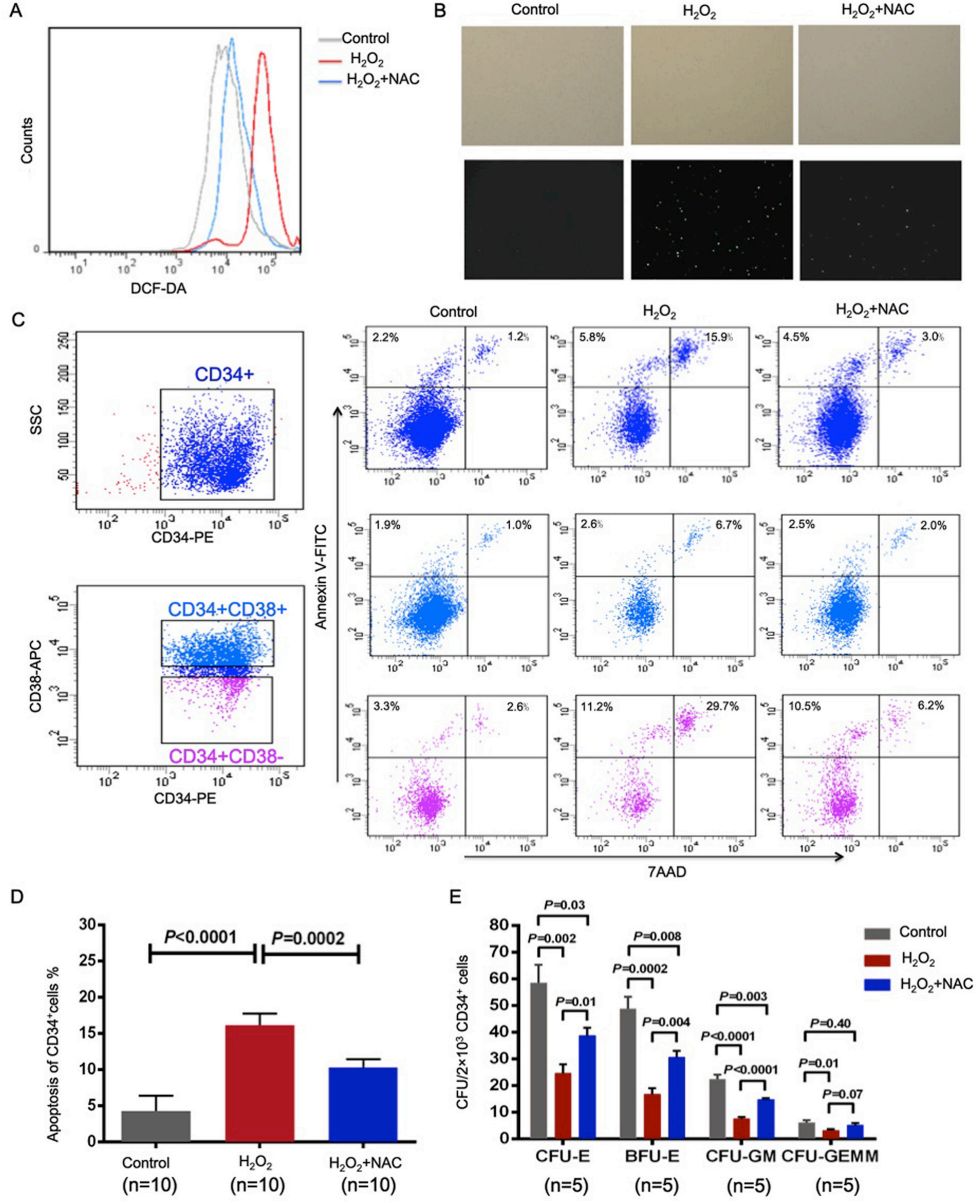
H_2_O_2_-induced increases in ROS that result in defective CD34-positive cells are partially reversed by N-acetyl-L-cysteine To further investigate the effect of oxidative stress on normal post-transplant hematopoiesis, CD34-positive bone marrow cells from subjects with good graft function were treated with H_2_O_2_ with or without N-acetyl-L-cysteine. The cells were then stained with DCFH-DA and analyzed by flow cytometry **A.** or fluorescence microscopy **B.** to detect intracellular ROS. H_2_O_2_ treatment of CD34-positive bone marrow cells from subjects with good graft function dramatically increased the percentage of apoptosis cells, whereas N-acetyl-L-cysteine partially but significantly restored apoptosis **C, D.** and the defective colony-forming unit plating efficiency **E.** of CD34-positive bone marrow cells.

Next, CD34-positive bone marrow cells from subjects with good graft function were incubated with H_2_O_2_ to simulate increased ROS model *in vitro* (Figure 6A, 6B). Higher levels of apoptosis were observed in the H_2_O_2_ group when compared with normals (Figure 6C, 6D, 16.13%±5.13% *vs*. 4.19%±1.00%; *P*<0.0001). Co-incubation with N-acetyl-cysteine partially reversed the frequency of apoptosis compared with the H_2_O_2_ group (10.28%±3.65% *vs.* 16.13%±5.13%; *P*=0.0002).

The CFU plating efficiencies (Figure 6E), including CFU-E (58±7 *vs*. 24±3; *P*=0.002), BFU-E (49±4 *vs.* 17±2; *P*=0.0002), CFU-GM (22±2 *vs*. 8±0.7; *P*<0.0001) and CFU-GEMM (6±1 *vs*. 3±0.5; *P*=0.01), of CD34-positive bone marrow cells from subjects with good graft function decreased significantly when the cells were incubated with H_2_O_2_. The addition of N-acetyl-L-cysteine partially reversed this effect for CFU-E (25±3 *vs*. 39±3; *P*=0.01), BFU-E (17±2 *vs.* 31±3; *P*=0.004), CFU-GM (8±0.7 *vs*. 15±0.6; *P*<0.0001) and CFU-GEMM (3±0.5 *vs*. 5±0.8; *P*=0.07).

### CD34-positive bone marrow cells from the donors of poor or good graft function subjects demonstrated comparable haematopoietic reconstitution capacity in the xeno-transplanted NOD-Prkdc^scid^IL2rg^null^ mice

**Figure 7:**
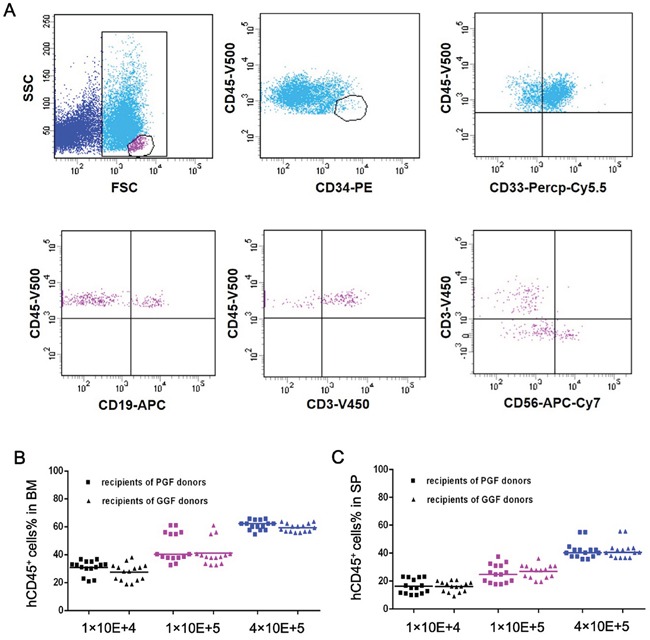
Haematopoietic reconstitution activity in NOD-Prkdc^scid^IL2rg^null^ mice is similar in bone marrow CD34-positive cells obtained from donors to subjects with poor or good graft function **A.** Representative immunophenotypic analyses of engrafted human CD45-positive cells, including CD34-positive cells and CD33-positive cells, NK cells (CD56-positive), B-cells (CD19-positive) and T-cells (CD3-positive) were shown. **B, C.** Engraftment levels in bone marrow and spleens from xeno-grafted NOD-Prkdc^scid^IL2rg^null^ mice were similar for bone marrow CD34-positive cells obtained from donors to subjects with poor or good graft function.

Bone marrow and spleen from all xeno-grafted NOD-Prkdc^scid^IL2rg^null^ mice contained large numbers of human haematopoietic cells which correlated with the dose of cells injected (Figure 7). Engraftment levels were similar for bone marrow CD34-positive cells obtained from donors of poor or good graft function subjects. Detailed analyses showed human CD34- positive cells and CD33-positive cells, NK cells (CD56-positive), B-cells (CD19-positive) and T-cells (CD3-positive).

### Intracellular ROS levels and cell cycle status in subjects with poor or good graft function were compared with those in their paired donors' bone marrow cells

**Figure 8:**
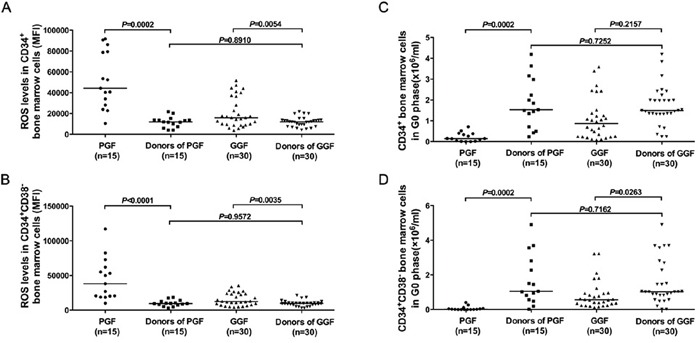
Intracellular ROS levels and cell cycle status in subjects with poor or good graft function were compared with those in their paired donors' bone marrow cells Although intracellular ROS levels and cell cycle status in CD34-positive **A, C.**, and CD34-positive, CD38-negative **B, D.** fractions of subjects with poor or good graft function were significantly different from those in their paired donors' bone marrow cells, similar intracellular ROS levels and cell cycle state were shown in CD34-positive (A, C), and CD34-positive, CD38-negative (B, D) fractions from donors of poor or good graft function subjects.

The intracellular ROS levels in CD34-positive (51820±7001 *vs.* 11903±1330; *P*=0.0002), and CD34-positive, CD38-negative cell fractions (44475±7752 *vs.* 10016±1107; *P*<0.0001) of subjects with poor graft function were significantly higher than those in their paired donors (Figure 8A, 8B). Moreover, the frequencies of quiescent cells in CD34-positive (0.22±0.06×10E+6 *vs*. 1.89±0.31×10E+6; *P*=0.002), and CD34-positive, CD38-negative cell fractions (0.07±0.03×10E+6 *vs*. 1.69±0.37×10E+6; *P*=0.0002) of subjects with poor graft function were significantly lower than those in their paired donors (Figure 8C, 8D).

However, the intracellular ROS levels of transplanted donors' CD34-positive (11903±1330 *vs.* 11915±827.9; *P*=0.8910), and CD34-positive, CD38-negative (9835±759.6 *vs.* 10016±1107, *P*=0.7252) for poor or good graft function subjects showed no significant differences (Figure 8A, 8B). Moreover, cell cycle state analyses demonstrated that the quiescent cells number of the transplanted donors' CD34-positive (1.85±0.31×10E+6 *vs.* 1.72±0.17×10E+6; *P*=0.9572), and CD34-positive, CD38-negative (1.69±0.37×10E+6 *vs*. 1.52±0.23×10E+6; *P*=0.7162) for subjects with poor or good graft function were similar (Figure 8C, 8D).

## DISCUSSION

Poor graft function following allotransplant remains a life-threatening complication with limited effective treatment options [1–4]. Several risk factors, including the number of transplanted CD34-positive cells, disease state, drug-induced toxicity, G*v*HD, infections, and an impaired bone marrow microenvironment have been reported to be associated with the occurrence of poor graft function [2, 3, 5–9]. However, no study has focused on the functional characterization of bone marrow CD34-positive cells in subjects with poor graft function following allotransplant or in donors to subjects with poor graft function pretransplant. Moreover, the effect of increased ROS on bone marrow CD34-positive cells in subjects with poor graft function following allotransplant is unclear.

Oxidative stress, in particular ROS, regulates haematopoietic stem and progenitor cells function in mice [14–18] and *Drosophia* [19]. Cells with low ROS levels have better long-term repopulating capacity compared with those with high ROS levels which are mostly involved with short-term repopulation [15]. Under normal conditions, haematopoietic stem and progenitor cells are found in hypoxic bone marrow microenvironment, a setting which protects them from oxidative stress [15–17]. In contrast, exceedingly high ROS production occurs under various pathological conditions, which can inhibit haematopoietic stem and progenitor cells self-renewal and induce DNA damage and apoptosis resulting in premature exhaustion of haematopoietic stem and progenitor cells and haematopoietic dysfunction [18, 20, 21].

Appropriate control of quiescence is crucial for normal haematopoietic stem and progenitor cells function [22–24]. Cell cycle changes affect the repopulating ability of murine stem cells [25–27]. We found haematopoietic stem and progenitor cells are functionally impaired in subjects with poor graft function and had a significantly lower fraction of quiescent bone marrow-derived CD34-positive cells compared with subjects with good graft function and with normals. However, it should be noted that the median age of the normal cohort is younger than those in the cohorts of poor graft function and good graft function in the current study. Our data are consistent with the hypothesis that poor graft function is associated with a defect in maintenance of haematopoietic stem and progenitor cells quiescence, which is in accordance with the worldwide practice that the administration of a CD34-positive selected stem cell boost is an effective option for improving graft function [1, 28–30].

Our data indicate impaired haematopoietic stem and progenitor cells function is associated with increased intracellular levels of ROS. This was associated with increased levels of p53, p21 but not p38, in contrast to the results of previous study [18]. Although whether the ROS elevation is the cause or consequence of poor graft function and the underlying molecular mechanisms remain to be clarified, our data provide evidence that elevated intracellular ROS lead to increased DNA damage and apoptosis *via* the p53-p21 pathway.

The sources and regulation of abnormal intracellular ROS in bone marrow CD34-positive cells from subjects with poor graft function have yet to be elucidated. Effective cross-talk between haematopoietic stem and progenitor cells and the bone marrow microenvironment is important for the regulation of haematopoiesis [10–13]. At the junction of these types of regulation, ROS produced endogenously *via* cellular respiration or nicotinamide adenine dinucleotide phosphate-oxidase activity (haematopoietic stem and progenitor cell-derived) [31, 32] as well as after exposure to exogenous stress (bone marrow microenvironment-derived) [16–18, 33] play important roles in regulating haematopoietic stem and progenitor cell functions. We previously reported that the bone marrow endosteal and vascular microenvironment are impaired in poor graft function post-transplant [2, 3]. In the current study, CD34-positive bone marrow cells from the donors to subjects with poor or good graft function exhibited comparable haematopoietic reconstitution capacities in the xeno-transplanted NOD-Prkdc^scid^IL2rg^null^ mice. Based on our data and previous reports [2, 3, 16–18, 33–35], it is conceivable that the preconditioning and some post-transplant events, such as G*v*HD, CMV reactivation, and some cytotoxic agents, may trigger abnormally increased ROS in bone marrow microenvironment, which may subsequently lead to defective haematopoietic stem and progenitor cells and the occurrence of poor graft function post-allotransplant. We are aware, however, that further studies are needed to determine whether the potential sources of the ROS are intrinsic to haematopoietic stem and progenitor cells, the bone marrow microenvironment, or both in the future.

In conclusion, although requiring further functional validation, our data demonstrated for the first time that even if the transplanted donors' bone marrow CD34-positive cells are functionally normal pretransplant, elevated ROS in bone marrow CD34-positive cells post-allotransplant may result in increased levels of DNA strand breaks, apoptosis, up-regulated levels of p53, p21 (but not p38), and exhaustion of quiescence in subjects with poor graft function. Moreover, H_2_O_2_-induced increases in ROS resulted in defective CD34-positive cells is partially reversed by N-acelyl-L-cysteine. Thus, our data suggest that it would be of value to investigate whether antioxidant drugs can benefit subjects with poor graft function post-allotransplant in phase I/II clinical trials in the future.

## MATERIALS AND METHODS

### Subjects and normals

Three cohorts were analyzed, subjects with poor or good graft function posttransplant and normals (transplant donors). Transplant recipients were identified from consecutive subjects receiving an allotransplant for a haematologic neoplasm April 1, 2014 to March 31, 2015 at Peking University Institute of Hematology and willing to participate in the study. 15 subjects developing poor graft function were eligible. For each case, 2 matched transplant recipients with good graft function were randomly-selected from the same cohort after matching for age, pretransplant disease state and interval posttransplant (“risk-set sampling”) [36]. Variables of subjects and controls are summarized in Table 1. Bone marrow samples from donors (n=45) were controls. The normal cohort comprised 25 males and 20 females, ages 18-55 years (median, 29 years). The study was approved by the Ethics Committee of Peking University People's Hospital, and written informed consent was obtained from all subjects compliant with the Declaration of Helsinki.

**Table 1:** Characteristics of allotransplants subjects with poor or good graft function

Characteristics	Poor Graft Function cases^a^ (n=15)	Good Graft Function cases^a^ (n=30)	*P*-Value^b^
BM evaluated time (post-HSCT days)	95(90-150)	93(90-148)	0.79
Blood cell count			
Median WBC (×10^9^/L) (range)	1.36(1.02-2.34)	4.40(1.99-6.05)	<0.0001
Median ANC (×10^9^/L) (range)	0.32(0.11-0.45)	3.24(0.9-5.15)	<0.0001
Median Hb (g/L) (range)	58(45-70)	103(76-125)	<0.0001
Median PLT (×10^9^/L) (range)	12(8-19)	102(45-243)	<0.0001
Age at HSCT (years, median, range)	42.5(18-54)	45.0(18-61)	0.79
Gender (male/female)	6/9	11/19	0.83
Underlying disease			
AML	7	15	0.83
ALL	5	9	0.82
MDS	3	6	1.00
Status at HSCT			0.66
Standard-risk	6	10	
High-risk	9	20	
Source of stem cell			1.00
BM and PB	15	30	
Transplanted total nucleated cell dose(×10^8^/kg, median, range)	7.18(4.23-9.68)	7.02(3.50-9.08)	0.55
Transplanted CD34^+^ cell dose(×10^6^/kg, median, range)	2.52(1.56-4.98)	2.43(1.23-5.02)	0.78
Donor match			0.81
HLA-identical sibling donor	4	7	
HLA-partially matched related donor	11	23	
Sex mismatch			
Female to male	2	5	0.77
Female to female	2	5	0.77
male to female	6	11	0.83
male to male	5	9	0.82
ABO mismatch			
No	8	15	0.83
Minor	5	10	1.00
Major	2	5	0.77
Pre-HSCT cycles of chemotherapy	3 (0-5)	4(0-7)	0.67
Conditioning			0.81
BU/CY	4	7	
BU/CY+ATG	11	23	
History of G*v*HD	9	19	0.83
Onset of aG*v*HD(days, median, range)	30(24-52)	28(23-49)	0.78
History of CMV reactivation	11	21	0.82
Onset of CMV reactivation(days, median, range)	27(20-46)	26(21-43)	0.86
CMV reactivation treated with ganciclovir	6	13	0.83

a Group matching criteria included age at HSCT (±1years), pre-HSCT cycles of chemotherapy (±1cycle), disease status at HSCT and BM microenvionment evaluated time after HSCT(±5 days). For each case, two GGF controls were randomly selected from the same cohort at which the PGF occurred (‘risk-set sampling’).

b The continuous variables were compared using the Mann-Whitney U-test, and the differences in frequency between the 2 groups were compared using the chi-square test. The criterion for statistical significance was *P*<0.05.

Abbreviations: allo-HSCT indicates allogeneic haematopoietic stem cell transplantation; PGF, poor graft function; GGF, good graft function; BM, bone marrow; PB, peripheral blood; WBC, white blood cell; ANC, absolute neutrophil cell; Hb, hemoglobin; PLT, platelet; AML, acute myelogenous leukemia; ALL, acute lymphocytic leukemia; MDS, myelodysplastic syndrome; HLA, human leukocyte antigen; G*v*HD, graft-versus-host disease; aG*v*HD, acute G*v*HD; CMV, cytomegalovirus.

### Clinical definitions and evaluation

Transplant recipients had to have complete donor haematological chimerism (see below) with no residual or recurrent leukaemia. Good graft function [2, 3] was defined as a continued engraftment (neutrophils >0.5×10E+9/L for 3 consecutive days, platelets >20×10E+9/L for 7 consecutive days without platelet transfusions and hemoglobin concentration >70 g/L without RBC transfusions) >28 days posttransplant. Poor graft function [2–4] was defined as a hypo- or aplastic bone marrow with 2 or 3 of the following: (1) neutrophils ≤0.5×10E+9/L; (2) platelets ≤20×10E+9/L; and/or (3) hemoglobin concentration ≤70 g/L for ≥3 consecutive days after day +28 posttransplant without platelet and/or RBC transfusion and/or G-CSF therapy.

Chimerism analyses were done by DNA fingerprinting for short tandem repeats in blood samples and/or by chromosome fluorescent *in situ* hybridization of bone marrow samples. Complete donor chimerism was defined as no recipient haematopoietic or lymphoid cells detected (sensitivity >0.1% recipient signals) [37].

Diseases were categorized as standard- or high-risk. Standard-risk was defined as 1^st^ or 2^nd^ complete remission (CR1 or CR2) of acute leukemia or myelodysplastic syndrome (MDS). All other subjects were classified as high-risk. Haematologic relapse was defined as blasts >5% in the blood, bone marrow or extra-medullary site. G*v*HD was scored as acute or chronic as previously described [2, 3, 37].

### Transplantation protocols

Donor selection, HLA-typing, graft harvesting, conditioning therapy and G*v*HD prophylaxis were done as reported [2, 3, 37, 38]. The subjects were screened pre-transplant for cytomegalovirus (CMV) infection by serology. Weekly real-time quantitative PCR was used to detect CMV reactivation in blood samples. CMV infections were treated with ganciclovir or foscarnet. After allotransplants, rhG-CSF (5 μg/kg/day) was administered to recipients of HLA-mismatched related transplants from day +6 until neutrophils were >0.5 × 10E+9/L for 3 consecutive days. rhG-CSF was not administered to recipients of HLA-identical sibling transplants, except in cases where neutrophils were <0.5 × 10E+9/L until day +21. The subjects received RBCs if their hemoglobin concentrations were ≤70 g/L, or following platelet transfusion if their platelets were ≤20 × 10E+9/L.

### Cell cycle and apoptosis analyses

Bone marrow mononuclear cells were isolated by density centrifugation using lymphocyte separation medium (GE Healthcare, Milwaukee, WI, USA). Cell cycle analyses were performed by incubating with 10 μg/ml Hoechst 33342 (Thermo Fisher Scientific, Waltham, MA, USA) at 37°C for 45 min, and 1.0 μg/ml Pyronin Y (Sigma, St. Louis, MO, USA) at 37°C for an additional 15 min. Cells were stained with mouse anti-human CD34-PerCP-Cy5.5 and CD38-APC-conjugated monoclonal antibodies (Becton Dickinson) at room temperature for 15 min.

To detect apoptosis, cells were incubated with CD34-PE, CD38-APC and CD45-V500 and then incubated for 15 min with Annexin-V-FITC and 7-amino-actinomycin D (7-AAD) apoptosis detection kit (Becton Dickinson) according to the manufacturer's instruction. Multi-parameter flow cytometric analyses were done using a BD LSRFortessa (Becton Dickinson). Aliquots of unstained samples were used as negative controls. Data were analyzed using BD LSRFortessa software (Becton Dickinson).

### Measurement of intracellular ROS levels

ROS staining was done using an ROS staining kit (Byotimes, Shanghai, China) according to the manufacturer's protocol. Cells were incubated with 10μM 2′,7′-dichlorofluorescence diacetate (DCFH-DA, Byotimes) and mouse anti-human CD45-V500, CD34-PerCP-Cy5.5, CD38-APC-conjugated monoclonal antibodies (Becton Dickinson) at 37°C for 15 min. After crossing the cell membrane, DCFH-DA undergoes de-acetylation by intracellular esterase producing green fluorescent when oxidized by ROS. Mean fluorescence intensity of intracellular ROS was analyzed as intracellular ROS levels using BD LSRFortessa software (Becton Dickinson).

### Measurement of intracellular proteins

1×10E+6 bone marrow mononuclear cells were incubated with mouse anti-human CD34-PerCP, CD38-APC-conjugated monoclonal antibodies (Becton Dickinson) at 37°C for 15 min then fixed, permeabilized, and incubated with phospho-Histone H2A.X-PE, phospho-p53-PE, p53-Alexa Fluor 488, p21-Alexa Fluor 647 and phospho-p38 MAPK-Alexa Fluor 647 (Cell Signaling Technology, Danvers, MA, USA). Levels were expressed as mean fluorescence intensity (mean ± SEM). We used the CaspGLOW fluorescein active caspase-3/caspase-9 staining kit (Biovision, Mountain View, CA, USA) and CD34-PerCP and CD38-APC-conjugated monoclonal antibodies (Becton Dickinson) according to the manufacturer's instructions.

### Colony-forming unit (CFU) assays

CD34-positive cells were isolated from the bone marrow mononuclear cells of subjects with poor or good graft function and their donors using CD34 MicroBead Kit (Miltenyi Biotec, Bergisch Gladbach, Germany). Purity of each subset was >92%. CFU were assayed using MethoCult™ H4434 Classic (Stem Cell Technologies, Vancouver, BC, Canada). 2×10E+3 CD34-positive cells were plated in 24-well plates and cultured for 14 days. Burst-forming unit erythroid (BFU-E), colony-forming unit granulocyte–macrophages (CFU-G-M), colony-forming unit erythroid (CFU-E), and colony-forming unit-granulocyte, -erythroid, -macrophage and -megakaryocyte (CFU-GEMM) were scored using an inverted light microscope. Cultures were done in triplicate and results expressed as mean ± SEM.

### Single-cell gel electrophoresis (the comet assay)

Sorted CD34-positive bone marrow cells were suspended in 25μl of 0.6% (w/v) low melting point agarose in PBS, and immediately pipetted onto a frosted glass microscope slide precoated with a layer of 0.8% (w/v) normal melting point agarose similarly prepared in PBS. The slides immersed in lysis solution at 4°C overnight to remove cellular protein. The slides were placed in electrophoresis tank containing 0.3 M NaOH and 1 mM EDTA for 30 min before electrophoresis at 28 V for 25 min at an ambient temperature of 4°C. The slides were then washed 3 times with 0.4 M Tris-HCl, pH 7.5, at 4°C before staining with 8μl DAPI. One hundred comets from each slide were electronically captured using fluorescence microscope, and the images were analyzed with CASP1.2.3 software (Institute of Theoretical Physics, University of Wroclaw, Wroclaw, Poland)

### Treatment of CD34-positive bone marrow cells with H_2_O_2_ and N-acetyl-L-cysteine

CD34-positive bone marrow cells were cultured with StemSpan™ SFEM (Stem Cell Technologies) supplemented with 50 ng/ml cytokines(SCF, FLT-3, and TPO) (PeproTech, Locky Hill, NJ, USA) and maintained at 37°C in a 5% CO_2_ atmosphere. There were too few cells from subjects with poor graft function for these experiments.

To further investigate the effect of oxidative stress on normal posttransplant haematopoiesis, CD34-positive bone marrow cells from subjects with good graft function were treated with 100 μM H_2_O_2_ (Sigma), for 24 h at 37°C with or without 1 mM N-acetyl-L-cysteine (Sigma). Quantification of the apoptosis, intracellular ROS levels and cell-cycle state of different groups were analyzed by flow cytometry (see above).

### Evaluation of the hematopoietic reconstituting activity of donor CD34-positive bone marrow cells in NOD-Prkdc^scid^IL2rg^null^ mice

NOD-Prkdc^scid^IL2rg^null^ mice were purchased from Beijing Vitalstar Biotechnology Co., Ltd., Beijing, China. The mice were maintained in specific pathogen-free conditions and fed sterilized water and food at the animal facility of Peking University People's Hospital. The mice were 4-6 weeks old at the time of transplantation. All animal experiments were approved by the Ethics Committee of Peking University People's Hospital.

CD34-positive cells were isolated from the bone marrow mononuclear cells of donors to subjects with poor or good graft function using a CD34 MicroBead Kit (Miltenyi Biotec). The hematopoietic reconstituting activity of the donor CD34-positive bone marrow cells was evaluated using a NOD-Prkdc^scid^IL2rg^null^ xenograft assay by intra-bone marrow injection [39, 40]. Briefly, mice received 1.5 Gy of total body irradiation from a ^60^Co source for 90 s. Within 24 h, intra-bone marrow injections of 1×10E+4, 1×10E+5 or 4×10E+5 CD34-positive bone marrow cells from donors to subjects with poor (n=5) or good graft function (n=5) were performed in triplicate.

Blood was obtained from the retro-orbital plexuses of the injected mice at 1, 3 and 6 mo. The mice were then euthanized at 6 mo, and the bone marrow, spleen and blood cells were evaluated for human hematopoietic reconstitution using mouse anti-human CD45-V500, CD34-PE, CD38-FITC, CD3-V450, CD4-PE-Cy7, CD19-APC, CD33-PerCP-Cy5.5 and CD56-APC-Cy7-conjugated monoclonal antibodies (Becton Dickinson). Human engraftment was quantified by the frequency of hCD45-positive cells.

### Statistical analyses

Statistical analyses were done using one-way ANOVA to compare the three groups. Subject variables were compared using the χ^2^ test for categorical variables and the Mann–Whitney U test for continuous variables. Intracellular ROS levels and cell cycle status in subjects with poor or good graft function were compared with those in their paired donors' bone marrow cells by paired-T test. Correlations between hematologic parameters and ROS levels in CD34-positive bone marrow cells in patients posttransplant were determined using Pearson correlation coefficients. Analyses were performed using SPSS 22.0 (IBM, Armonk, NY, USA) and GraphPad Prism 6.0 software (GraphPad Software, La Jolla, CA, USA) packages, and *P*-values < 0.05 were considered statistically significant.
